# Hydroxyurea and sickle cell anemia: effect on quality of life

**DOI:** 10.1186/1477-7525-4-59

**Published:** 2006-08-31

**Authors:** Samir K Ballas, Franca B Barton, Myron A Waclawiw, Paul Swerdlow, James R Eckman, Charles H Pegelow, Mabel Koshy, Bruce A Barton, Duane R Bonds

**Affiliations:** 1 Cardeza Foundation, Department of Medicine, Jefferson Medical College, Philadelphia PA, USA; 2 Maryland Medical Research Institute, Baltimore MD, USA; 3 National Heart, Lung, and Blood Institute, National Institutes of Health, Bethesda MD, USA; 4 Wayne State University Detroit MI, USA; 5 Emory University, Atlanta GA, USA; 6 University of Miami, Coral Gables FL, USA; 7 University of Illinois at Chicago Hospital, Chicago IL, USA

## Abstract

**Background:**

The Multicenter Study of Hydroxyurea (HU) in Sickle Cell Anemia (MSH) previously showed that daily oral HU reduces painful sickle cell (SS) crises by 50% in patients with moderate to severe disease. The morbidity associated with this disease is known to have serious negative impact on the overall quality of life(QOL) of affected individuals.

**Methods:**

The data in this report were collected from the 299 patients enrolled in the MSH. Health quality of llife (HQOL) measures were assessed in the MSH as a secondary endpoint to determine if the clinical benefit of HU could translate into a measurable benefit perceptible to the patients. HQOL was assessed with the Profile of Mood States, the Health Status Short Form 36 (SF-36), including 4-week pain recall, and the Ladder of Life, self-administered twice 2-weeks apart pre-treatment and every 6 months during the two-year, randomized, double-blind, treatment phase. The effects of factors including randomized treatment, age, gender, pre-treatment crises frequency, Hb-F level mean, daily pain from 4-week pre-treatment diaries, and 2-year Hb-F response level (low or high) were investigated.

**Results:**

Over two years of treatment, the benefit of HU treatment on QOL, other than pain scales, was limited to those patients taking HU who maintained a high HbF response, compared to those with low HbF response or on placebo. These restricted benefits occurred in social function, pain recall and general health perception. Stratification according to average daily pain prior to treatment showed that responders to HU whose average daily pain score was 5–9 (substantial pain) achieved significant reduction in the tension scale compared to the placebo group and to non-responders. HU had no apparent effect on other QOL measures.

**Conclusion:**

Treatment of SS with HU improves some aspects of QOL in adult patients who already suffer from moderate-to-severe SS.

## Background

Health-related quality of life (HQOL) is a multidimensional concept that includes the physical, emotional, and psychosocial components associated with a disease or its treatment [1] in different domains of life [2-4], addressing the patients' perceptions of their situations [2-4]. Increasing self-reported satisfaction in the domains of life is associated with higher levels of quality of life. Improving HQOL has become an important objective of medical care [2-4]. It is the goal of health care providers to enhance treatment outcome and restore comfort and well being to the lives of their patients. Achievement of this goal, however, is a function of the disease process that is being treated. Thus, improving the HQOL of patients with chronic disease and of patients with chronic pain is a challenge.

Acute episodes of pain are the principal symptom of sickle cell disease (SCD). In the Cooperative Study of Sickle Cell Disease (CSSCD), an analysis of painful crises in 3,578 patients ranging in age from newborns to age 66 [5-7] conducted from ***1982–1995***, the average crisis rate was 0.8 episodes per patient-year in SS, 1.0 episode per patient-year in sickle β^0^-thalassemia and 0.4 episodes per patient-year in SC and sickle β^+^-thalassemia. The rates varied widely within each of these 4 groups: 39% of patients with sickle cell anemia had no episodes of pain, and 1% had more than 6 episodes per year. The 5.2% of CSSCD patients with 3 to 10 episodes per year had 32.9% of all episodes. Among CSSCD patients over the age of 20, those with high crisis rates had higher mortality levels than those with low crisis rates. High crisis rates were associated with high hematocrit and low fetal hemoglobin levels. Fetal hemoglobin level was predictive of the crisis rate, suggesting that attempts to increase the fetal hemoglobin level with pharmacological agents such as hydroxyurea might decrease the crisis rate and ultimately improve survival. CSSCD data suggest that sickle cell disease can have an impact on the patient's ability to complete education, develop work skills and hold full-time employment. SCD can influence social and family adjustment also.

The Multicenter Study of Hydroxyurea in Sickle Cell Anemia (MSH) Trial was a randomized, double-blind, placebo (PL)-controlled trial conducted in 299 SS patients with 3 or more annual crises at 21 clinical centers [8,9]. Daily oral hydroxyurea reduced painful sickle cell episodes, hospitalizations for painful episodes, acute chest syndrome and total number of units of blood transfused.

Although patients with sickle cell anemia (SS) have been treated with opioid and non-opioid analgesics routinely in an effort to decrease their pain and suffering, there has been little information on the effect on QOL from controlled trials to reduce pain in this patient population.

As part of the MSH, demographic and quality of life measures were obtained from all study subjects to assess the impact of hydroxyurea therapy on the patients' perception of pain, stamina and the day-to-day activities of living. We report on the effect of treatment of SS anemia on QOL. This is a secondary endpoint analysis for randomized treatment with hydroxyurea.

## Methods

The patients included in this report are the 277 of the 299 enrolled in the Multicenter Study of Hydroxyurea in Sickle Cell Anemia (MSH) [8,9] conducted from 1992 to 1995. Daily oral HU was titrated to maximum doses that did not cause unacceptable marrow depression over an average 2.4 years after randomization. The health related quality of life measures (QOL) collected twice 2 weeks apart pre-treatment (the mean was used as the baseline value) and every 6 months up to 2 years of randomized treatment were the MOS Health Status Survey Short Form (SF-36), the Profile of Mood Status (POMS) and the ladder of life [10-13]. Use of the MOS Short Form 36 General Health Survey was by courtesy of the Medical Outcomes Institute. Patients completed diaries of daily bodily pain rated on a 10-step scale with 0 = no pain and 9 = worst imaginable pain. Mean daily pain was computed over the 28 days pre-treatment a covariate for analysis.

Patients gave signed informed consent for participation in the randomized trial.

Health Status Survey (SF-36) results were scored according to the manual [11]; scores were converted to a 0–100% scale for ready comparison with results from other populations with 0 signifying the worst and 100 the best; for the SF-36 pain recall (distinct from the pain diary measure), 0 signified greatest pain (also worst) and 100, least pain. The domains measured with the Profile of Mood States were Tension-Anxiety and Depression-Dejection in which *lower *scores indicate *lower *tension or depression, i.e., better state, and Vigor and Fatigue in which *lower *scores indicate *worse *state (less vigor or more fatigue, respectively). The ladder of life is a subjective 10-step scale from "the best life for you" (level 10) to "the worst possible life for you" (level 1).

Only those patients with a 2-year Hb-F response (N = 277) were included in the analysis.). Of the remaining 22 patients, 6 had died by 2 years and the remaining 16 were no longer returning for clinic visits or did not give provide blood specimens for HbF determination (145). Of the 277, those missing a small number of HQOL data points during the 2-year follow-up comprised 1 additional HU who died, 3 HU patients lost to follow-up and 2 PL patients lost to follow-up. Death and loss to follow-up in the first 2 years resulted in HQOL data missing at random.

### Statistical methods

Health-related quality of life (HQOL) results for the MSH patients are presented as mean scores and standard errors in the Appendix tables and as mean ± 99% confidence interval in the figures, over time, according to HU- or PL-assigned randomized treatment, or grouped according to assigned treatment and HbF response at two years [14]. Differences between randomized groups (i.e., intention-to-treat analyses) and between post-hoc groups (i.e., high HbF responders vs. low responders by two years and placebo) are tested with generalized estimating equations models (GEE), which account for multiple (serial) observations per patient and consequent auto-correlation. Models were constructed starting with univariate models testing for the effects of treatment group assignment on the change from baseline to 6, 12, 18 and 24 months of each outcome measure. This initial model was expanded in a step-wise fashion to include other characteristics related to outcomes of the treatment that could potentially be related to QOL. Interaction tests were performed before combining first-order terms in more comprehensive models. Characteristics tested univariately first for their effect on QOL outcomes included pre-treatment QOL measures, gender, age, average daily pain from patient diaries, pre-treatment rate of acute vaso-occlusive (painful) crisis (<6/yr or ≥ 6/yr) and pre-treatment HbF (<0.5 g/dL or ≥ 0.5 g/dL). These factors were then included together with initial treatment assignment or HbF response in higher-order, multiply adjusted models.

Because of multiple testing, and the fact that QOL measures are considered secondary end points in the trial, the strength of statistical evidence for differences between groups was pre-determined at a more stringent level than the overall trial design of α = 0.05. The trial was not designed to detect specified differences in secondary measures *a priori*. Thus, observed differences carrying a nominal p-value of <0.01 are considered indicative of a possible difference, and those with a p-value of <0.001 are considered to provide stronger evidence of a difference.

## Results

**Figure 1:**
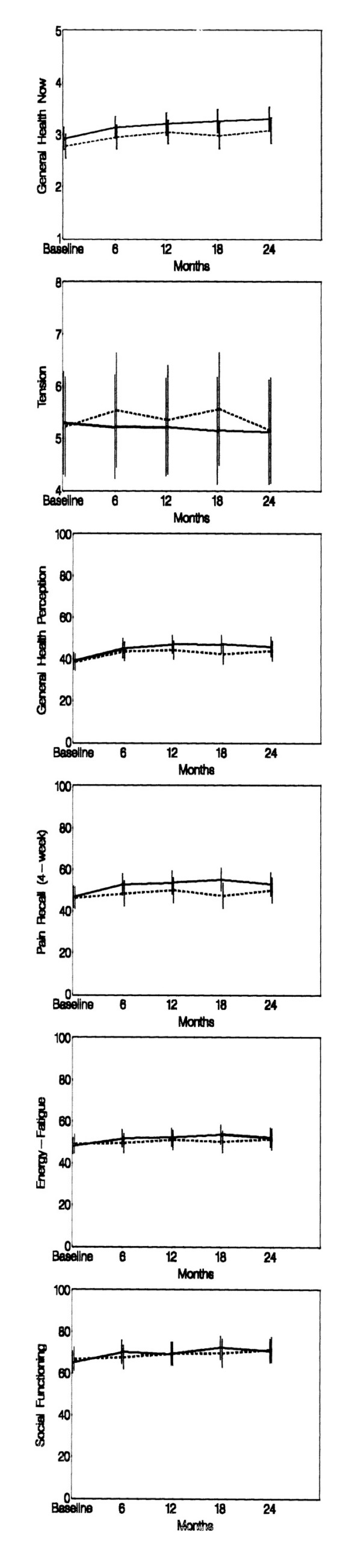
Selected parameters from MOS Short-Form SF-36 Health Quality of Life and the Profile of Mood States in adults with moderate-severe sickle cell anemia, treated with hydroxyurea (solid line) or placebo (dashed line) for two years. Mean quality of life measures and the standard error of the mean are shown.

Of the 299 MSH patients, 49% were male and 51% female. Their age distribution at trial entry was as follows: 18–29 (51%), 30–39 (39%), 40–49 (9%), >=50 (1%). Fifty-six percent had 6 or more crises annually (Fig 1A) and 44% reported mean daily pain of 4 or more on a scale of 0–9 (Fig 1B). The baseline HQOL results of the total MSH patient population were compared to published results including the Medical Outcomes Study [2], reference groups and a prospective cohort of sickle cell patients [15] followed at the Medical College of Georgia (MCG) 1985–1995 (Table 1). Both groups of sickle cell patients (MCG and MSH) had lower SF-36 scores on all measures, compared with a large reference group with no chronic conditions. MSH patients were quite comparable to the MCG sickle cell patients in all six measures that could be compared from the SF-36. They scored considerably worse than the reference group with the lowest SF-36 scores, namely patients with myocardial infarction, and also worse than the reference group with chronic pain, namely arthritis.

**Table 1:** Comparison of MSH patients and other reference patient groups on SF-36 Measures

	**Health Status Short Form 36 Scores**					
**Patient Group**	**Physical Functioning**	**Role-Physical**	**Social Functioning**	**Mental Health**	**Health Perception**	**Bodily Pain Recall**
No Chronic Conditions* (N = 2595)	86.0	87.2	92.3	77.6	72.6	74.2
Myocardial Infarction* N = 147	64.6	58.6	80.7	74.8	62.7	71.7
Arthritis* N = 2079	81.1	76.9	88.4	74.6	65.3	58.6
**Sickle Cell Anemia ^**† **^N = 143**	63.6	45.3	65.1	70.0	42.4	44.8
MSH** N = 299	66.0	46.7	6.65.0	70.0	38.8	46.5

*Data from Stewart et. al (2), † Data from Woods et. al (15), ** MSH: Multicenter Study of Hydroxyurea in Sickle Cell Anemia

**Figure 2:**
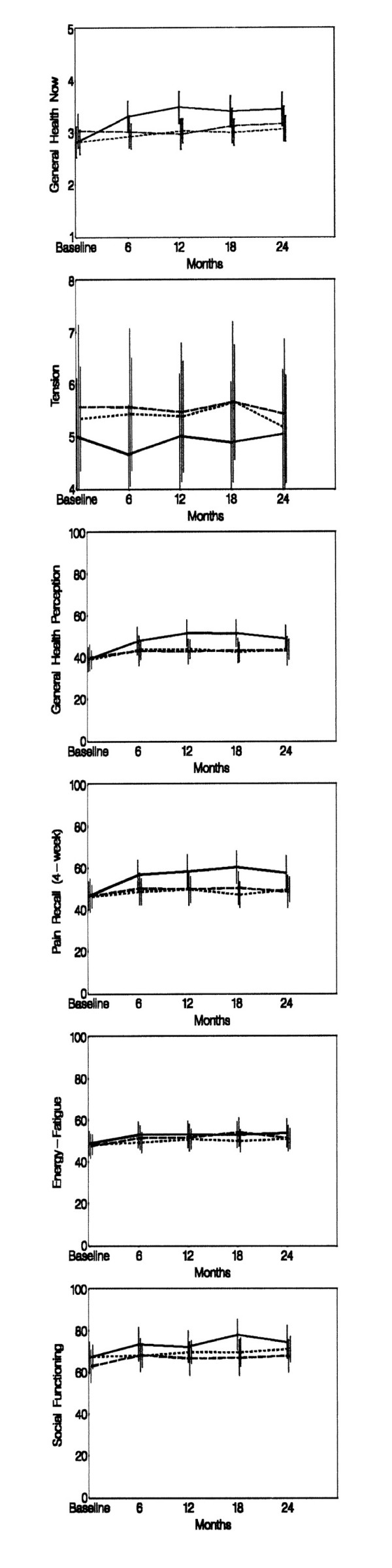
Selected parameters from MOS Short-Form SF-36 Health Quality of Life and the Profile of Mood States in adults with moderate-severe sickle cell anemia, treated with hydroxyurea or placebo for two years, and classified according to 2-year Hb-F response or placebo (short dashes). High responders (solid line) were above the 50^th ^percentile of HbF change from baseline to 2-years in the hydroxyurea group; low responders (long dashes) were below the 50^th ^percentile. Mean quality of life measures and the standard error of the mean are shown.

**Figure 3:**
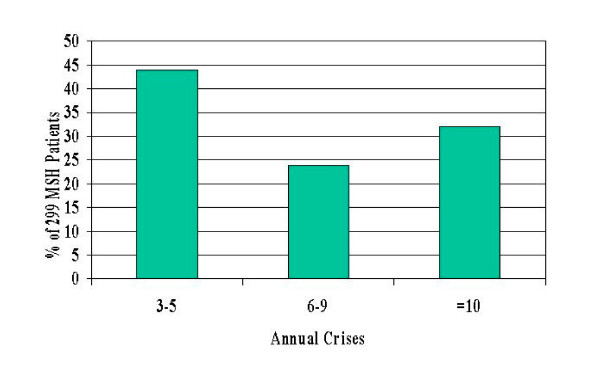
Distribution of number of annual crises in 299 MSH patients at trial entry.

Baseline QOL results were available on all 299 MSH patients according to originally assigned treatment group. In two years of follow-up, there were a few losses of patients to death (n = 8) and inability to complete follow-up visits (n = 16). Fig. 1 shows the mean ± 3 × SE of the HQOL measures according to original treatment group assignment and time (pre-treatment and every six months for two years of the trial). The data shown in Figs. 2 and 3 are listed in Tables A and B of the Appendix.

Analysis of daily pain and frequency of pain episodes show that these subjects were severely affected by sickle cell disease at study entry (Fig. 1). Mean daily pain on a 0- to-9 scale self-reported for four weeks pre-treatment, was distributed as follows: 7% reported zero mean daily pain, 49% reported pain averaging 1–3, 35% had average pain 4–6 and 9% reported average daily pain of 7–9 (Fig. 3A). Pre-treatment annual crisis rates were distributed as follows: 44% had 3–5 annual crises, 24% had 6–9 and 32% had 10 or more (Fig 1B).

At entry, 33% of patients had HbF >0.5 g/dl. At two-years, 49% of HU-assigned patients had >0.5 g/dl HbF (mean increase of 0.4 g/dl) compared to 23% of PL-assigned patients (mean loss of 0.1 g/dl). Of the HU-assigned patients defined as having the highest HbF response (the upper half in %HbF change), 79% had >0.5 g/dl HbF at two-years (mean increase of 0.8 g/dl Hb F), compared to 19% in low responders (zero mean change).

The PL and HU groups were comparable at baseline for all scales of QOL. Over two years of treatment, no differences between HU and PL in HQOL met the secondary endpoint critical value of p < 0.01 (Fig 3) However, HU-assigned patients with a high two-year response to HbF scored significantly better in "general health now" (p < 0.001), four-week pain recall (p = 0.004) and general health perception (p = 0.001) compared to those with low two-year HbF response or placebo (Fig 2 and Appendix Table B). A regression model of the effect of high HbF response on QOL adjusting for baseline values of QOL measures, HbF, annual crisis rate and mean daily pain are shown in Table 2. The baseline value of each QOL outcome was always predictive of the outcome at follow-up. The results of the model for each QOL outcome (columns) magnified the effect of high HbF response to HU on the four QOL outcomes – general health now (p < 0.001), general health perception (p < 0.001), 4-week pain recall (p < 0.001) and social functioning (p = 0.007), which was not statistically significant (p > 0.01) in a univariate model testing original treatment assignment only. Baseline daily pain was an independent predictor of the change in many of the QOL measures at all time points. Stratification by average daily pain shows that high HbF responders to HU whose average daily pain score was 5–9 achieved significant reduction in the tension scale compared to the placebo group and non-responders (p = 0.001, data not shown). However, HU showed no significant effect or trend of outcome difference on physical functioning, physical role and ladder of life. There were no significant time-by-treatment interactions.

**Table 2:** Multivariate generalized estimating equations (GEE) model to assess the effect of high HbF response on QOL. The effect of independent variables (rows) on each outcome measure (columns) at 6, 12, 18 and 24 months during randomized treatment to hydroxyurea or placebo is shown. The dependent variables are: SF-36 Health Status Survey variables (as a percent scores), Mood Profile, Ladder of Life.

**Independent Variables**	**quality of life Outcome Variables (each column is one model per outcome)**						
	General Health Now	Physical Functioning	Social Functioning	Role-Physical	Role-Mental	Mental Health	Energy-Fatigue
High Response	0.0008	NS*	0.007	NS	NS	NS	NS
Baseline QOL Value	<0.0001	< 0.0001	< 0.0001	< 0.0001	< 0.0001	< 0.0001	< 0.0001
Baseline Daily Pain 0–3, 4–6, 7–9	<0.0001	NS	<0.0001	<0.0001	0.008	0.002	0.002
Baseline Fetal Hemoglobin <0. 5 *vs *> 0. 5	NS	NS	NS	NS	NS	NS	NS
Baseline Annual Crisis Rate <6 *vs *≥ 6	0.002	NS	NS	0.0005	NS	NS	NS

	Pain Recall (4-week)	General Health Perception	Tension-Anxiety	Depression-Dejection	Vigor	Fatigue	Ladder of Life

High Response	0.0006	0.002	NS	NS	NS	NS	NS
Baseline QOL Value	< 0.0001	< 0.0001	< 0.0001	< 0.0001	<0.0001	<0.0001	< 0.0001
Baseline Daily Pain 0–3, 4–6, 7–9	< 0.0001	NS	0.001	<0.0001	NS	0.0004	0.007
Baseline Fetal Hemoglobin <0. 5 *vs *> 0. 5	NS	NS	NS	NS	NS	NS	NS
Baseline Annual Crisis Rate <6 *vs *≥ 6	<0.0001	0.003	NS	NS	0.004	NS	0.008

* NS = p > .01

## Discussion

Patients with chronic medical conditions (sickle cell anemia of any severity, Types I & II diabetes, congestive heart failure, myocardial infarction, arthritis, chronic lung problems, gastrointestinal disorders, back problems and angina) have worse physical role and social functioning, mental health, health perception, and/or body pain compared with patients with no chronic conditions [2,15,16]. Moreover, pain, by itself, has a negative impact on the financial, emotional, legal, familial, physical, social, occupational and behavioral dimensions of life [4]. Alleviation of pain by different modalities has been shown to improve the QOL for conditions other than sickle cell disease [17-20].

Hallmarks of SS include both recurrent episodes of acute pain as well as chronic pain. Woods et al [15] used the SF-36 to measure eight domains of health related to QOL in 143 adult SCD patients. Compared with patients with other chronic conditions, patients with SCD had lower (worse) scores than all other groups in the domains of physical role functioning, social functioning, health perception and body pain. In the domain of physical functioning, they had very low scores comparable to the scores of patients with congestive heart failure. Health and functional status scores for patients with SCD were similar to or lower than scores for patients with diabetes, congestive heart failure or back problems [[2,15-17] Table 1].

The impact of SCD on QOL is apparent in socioeconomic data on 3,538 African-American patients enrolled in the Cooperative Study of Sickle Cell Disease (CSSCD). That study found that a higher percentage of patients were unemployed and disabled compared to the US African-American population [6]. Fuggle et al [21] found that sickle cell pain resulted in over seven times increased risk of absenteeism from school and was highly disruptive of social and recreational activity of children with SCD. However, Kater et al [22], found that children with SCD in the Amsterdam area were compromised in the physical and psychological well-being, but not in cognitive or social aspects of QOL. Anie et al [23] reported that recurrent pain is not the only feature of SCD but other impairments in health-related quality of life are also important features. McClish et al [24] found that patients with SCD experience health-related quality of life that is worse than in the general population and most similar to patients undergoing hemodialysis. These studies [22-24], however, were not randomized, double-blind clinical trials, which MSH was.

Treatment of SS patients with HU improves their clinical and hematological characteristics [8,9,23,24]. The present data show that hydroxyurea can also improve some measures of QOL including "general health now," general health perception and pain recall. The effect was especially evident in patients with sustained HbF response to HU. The strength of the favorable effect on social functioning, however, was not significant (p > 0.01).

Lack of demonstrable effect of HU relative to PL on certain QOL measures in our patients (physical functioning, physical role and ladder of life) may be the result of issues inherent in the process of selection for the MSH of patients with moderate-to-severe disease who were already debilitated and had irreversible effects of their disease. Our patients had frequent acute pain episodes and high levels of mean pain scores reported at baseline (Fig. 1A and 1B). Even though many experienced an average of 50% reduction in acute pain episodes [8,9], they still had frequent acute pain episodes and high levels of chronic pain. Patients with complications like avascular necrosis may have irreversible limited range of motion of affected joints and patients with previous strokes may have permanent neurological defects. In our study there was strong evidence that these aspects of quality of life were influenced by the patients' quality of life, daily pain, and annual crises rate before enrollment (Table 2). Earlier treatment of individuals with SS before they have severe consequences of their disease may be an area for future investigation.

The absolute scores by the ladder of life scale are better than expected. This may reflect the optimism and positive attitudes of subjects who enrolled in this clinical trial. Subjects who consented to participate in the study may have been eager to benefit from the possibility of improvement that could be brought in by HU therapy; they received regular attention and clinical center staff support that may not be available to all patients with SCA.

Even though the high and low HbF responder analysis is post-hoc (i.e., based on 2-year HbF data after the QOL data was collected), the HbF responses occurs within weeks of HU initiation and is sustained through 2 years of treatment. Our data indicate that the increase in HbF level in subjects with SCD reduces the frequency of painful episodes [8,9] and now is shown to improve certain measures of HQOL. Because subjects in this study already had moderate-to-severe SS, improvements in QOL with HU treatment relative to PL were moderate and concentrated among those patients with high HbF response to HU. A question for further investigation is whether patients with less severe disease, treated with HU before they develop irreversible debilitating side effects, may benefit from prevention of loss of health status and quality of life.

## Conclusion

The quality of life of patients with sickle cell anemia is severely compromised similar to or even worse than patients with other chronic diseases such as arthritis. Patients who responded to hydroxyurea therapy had improvement in certain aspects of quality of life including social function, pain recall, and general health perception, in addition to the decrease in frequency of acute painful episodes, acute chest syndrome, and blood transfusion, responders to hydroxyurea with a pain score >5 achieved significant reduction in the tension scale compared to the placebo group and to non-responders.

## Competing interests

The author(s) declare that they have no competing interests.

## Authors' contributions

All authors participated in the design and coordination of the study. Samir K. Ballas and Franca B. Barton drafted the manuscript. Franca B. Barton, Myron A. Waclawiw, and Michael L. Terrin performed the statistical analyses and interpreted the results. James R. Eckman and Duane R. Bonds helped in editing the manuscript. All authors read and approved the final manuscript.

**Figure 4:**
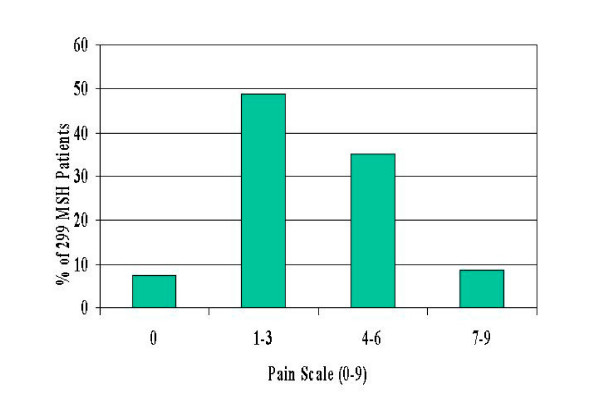
Distribution of average daily pain from two-week diaries at trial entry.

## Supplementary Material

Additional File 1 Ballas Appendix.doc. Tables A and B Click here for file
