# Supplementary material for: Hydroxyurea and sickle cell anemia: effect on quality of life
Source: Health Qual Life Outcomes. 2006 Aug 31;4:59. doi: 10.1186/1477-7525-4-59 (PMC1569824; doi:10.1186/1477-7525-4-59)
Supplement: Additional File 1 — Ballas Appendix.doc. Tables A and B [file 1477-7525-4-59-S1.doc]

##### Appendix

**Table A.** Results of Profile of Mood States (A-D), Ladder of Life (E) and MOS Short-Form SF-36 Health Quality of Life questions (F-N) in the MSH patients: 299 adults with moderate-severe sickle cell anemia, treated with hydroxyurea or placebo for two years. Mean quality of life measures and the standard error of the mean are shown.

**Hydroxyurea** **Placebo**

**Months** N Mean SE N Mean SE

A. Tension

0 152 5.3 0.4 147 5.2 0.4

6 148 5.2 0.4 137 5.5 0.4

12 146 5.2 0.4 138 5.4 0.4

18 144 5.2 0.4 136 5.6 0.4

24 141 5.1 0.4 136 5.2 0.4

B. Depression

0 152 4.9 0.5 147 5.0 0.5

6 148 5.1 0.5 137 5.0 0.5

12 146 5.2 0.5 138 5.0 0.6.

18 144 5.2 0.5 136 5.3 0.6

24 141 4.5 0.5 136 5.2 0.5

C. Vigor

0 152 9.3 0.4 147 9.1 0.4

6 148 9.8 0.4 137 9.1 0.4

12 146 10.1 0.4 138 9.7 0.4

18 144 10.4 0.4 136 9.6 0.5

24 141 9.9 0.4 136 9.8 0.5

D. Fatigue

0 152 6.1 0.3 147 6.5 0.4

6 148 5.9 0.4 137 6.0 0.4

12 146 5.9 0.4 138 6.2 0.4

18 144 5.7 0.4 136 6.3 0.4

24 141 5.3 0.4 136 6.0 0.4

E. Ladder of Life

0 152 6.7 0.2 147 6.3 0.2

6 148 6.8 0.2 137 6.5 0.2

12 146 6.8 0.2 138 6.8 0.2

18 144 6.7 0.2 136 6.8 0.2

24 141 6.7 0.2 136 6.7 0.2

**Hydroxyurea** **Placebo**

**Months** N Mean SE N Mean SE

F. General Health Now

0 152 2.9 0.1 147 2.8 0.1

6 148 3.1 0.1 137 3.0 0.1

12 146 3.2 0.1 138 3.0 0.1

18 144 3.3 0.1 136 3.0 0.1

24 141 3.3 0.1 136 3.1 0.1

G. General Health Perception

0 152 39.0 1.6 147 38.6 1.6

6 148 45.1 1.8 137 43.6 1.8

12 146 47.1 1.7 138 44.3 1.7

18 144 47.0 1.7 136 42.3 1.8

24 141 45.9 1.8 136 43.9 1.9

H. Physical Functioning

0 152 64.6 1.8 147 64.8 1.9

6 148 64.9 1.9 137 66.5 2.0

12 146 64.9 2.0 138 64.9 2.0

18 144 68.2 2.0 136 65.8 2.2

24 141 66.2 2.0 136 65.8 2.1

I. Social Functioning

0 152 65.2 2.1 147 66.8 2.2

6 148 70.3 2.2 137 67.7 2.2

12 146 69.5 2.1 138 69.5 2.2

18 144 72.4 2.2 136 69.8 2.6

24 141 70.9 2.2 136 71.4 2.4

J. Role‑Physical

0 152 48.4 3.4 147 45.1 3.6

6 148 59.6 3.4 137 51.5 3.5

12 146 52.2 3.5 138 50.7 3.7

18 144 55.9 3.5 136 50.6 3.7

24 141 51.6 3.6 136 49.4 3.7

K. Role‑Mental

0 152 70.4 3.2 147 66.7 3.3

6 148 69.1 3.1 137 67.9 3.2

12 146 61.0 3.4 138 63.5 3.6

18 144 62.5 3.5 136 68.6 3.4

24 141 68.8 3.4 136 62.5 3.7

**Hydroxyurea** **Placebo**

**Months** N Mean SE N Mean SE

L. Mental Health

0 152 71.5 1.4 147 71.3 1.6

6 148 70.0 1.6 137 70.2 1.6

12 146 70.2 1.6 138 71.9 1.6

18 144 70.0 1.7 136 71.4 1.7

24 141 70.6 1.7 136 71.5 1.6

M. Energy‑Fatigue

0 152 48.2 1.5 147 49.1 1.8

6 148 51.8 1.6 137 49.6 1.8

12 146 52.3 1.7 138 51.1 1.8

18 144 53.8 1.8 136 50.3 2.0

24 141 52.3 1.8 136 51.5 1.9

1. Pain Recall (4‑week)

0 152 46.8 2.0 147 46.3 2.0

6 148 52.8 2.0 137 48.3 2.4

12 146 53.7 2.2 138 50.0 2.3

18 144 55.1 2.2 136 47.3 2.4

24 141 53.0 2.2 136 50.0 2.3

**Table B.** Results of Profile of Mood States (A-D), Ladder of Life (E) and MOS Short-Form SF-36 Health Quality of Life questions (Panels F-N.) in the MSH patients: 299 adults with moderate-severe sickle cell anemia, treated with hydroxyurea or placebo for two years, and classified according to 2-year Hb-F response or placebo. High responders were above the 50^th^ percentile of HbF change from baseline to 2-years in the hydroxyurea group; low responders were below the 50^th^ percentile. Mean quality of life measures and the standard error of the mean are shown.

**High Responders Low Responders Placebo**

**Months** N Mean SE N Mean SE N Mean SE

A. Tension

0 71 4.9 0.5 72 5.6 0.6 134 5.3 0.4

6 70 4.4 0.5 71 5.6 0.6 130 5.4 0.4

12 70 4.8 0.5 71 5.5 0.5 133 5.4 0.4

18 70 4.6 0.6 71 5.7 0.6 133 5.7 0.4

24 69 4.9 0.5 70 5.4 0.6 133 5.1 0.4

B. Depression

0 71 3.7 0.5 72 5.8 0.9 134 5.1 0.6

6 70 4.0 0.7 71 5.6 0.7 130 4.9 0.5

12 70 4.4 0.7 71 6.0 0.8 133 5.0 0.6

18 70 3.9 0.7 71 6.5 0.8 133 5.4 0.6

24 69 3.7 0.5 70 5.1 0.8 133 5.2 0.5

C. Vigor

0 71 9.6 0.6 72 8.9 0.7 134 9.0 0.5

6 70 10.4 0.6 71 9.3 0.7 130 9.1 0.4

12 70 10.2 0.6 71 9.9 0.6 133 9.7 0.4

18 70 10.6 0.7 71 10.4 0.7 133 9.6 0.5

24 69 10.4 0.6 70 9.4 0.6 133 9.7 0.5

D. Fatigue

0 71 6.0 0.5 72 6.1 0.5 134 6.5 0.4

6 70 5.7 0.5 71 5.9 0.6 130 6.0 0.4

12 70 6.2 0.6 71 5.6 0.5 133 6.3 0.4

18 70 5.7 0.5 71 5.7 0.6 133 6.3 0.5

24 69 5.2 0.5 70 5.4 0.5 133 6.1 0.4

E. Ladder of Life

0 71 6.9 0.2 72 6.4 0.3 134 6.3 0.2

6 70 7.0 0.2 71 6.8 0.2 130 6.6 0.2

12 70 6.7 0.2 71 6.7 0.3 133 6.8 0.2

18 70 6.8 0.2 71 6.6 0.3 133 6.8 0.2

24 69 6.8 0.2 70 6.7 0.2 133 6.7 0.2

**High Responders Low Responders Placebo**

**Months** N Mean SE N Mean SE N Mean SE

F. General Health Now

0 71 2.8 0.1 72 3.0 0.1 134 2.8 0.1

6 70 3.3 0.1 71 3.0 0.1 130 2.9 0.1

12 70 3.5 0.1 71 3.0 0.1 133 3.0 0.1

18 70 3.4 0.1 71 3.1 0.1 133 3.0 0.1

24 69 3.4 0.1 70 3.2 0.1 133 3.1 0.1

G. General Health Perception

0 71 39.2 2.2 72 39.9 2.4 134 39.1 1.6

6 70 47.9 2.6 71 43.1 2.8 130 43.7 1.8

12 70 51.6 2.4 71 42.7 2.3 133 43.9 1.7

18 70 51.6 2.5 71 43.3 2.3 133 42.4 1.9

24 69 49.1 2.5 70 43.0 2.7 133 43.7 1.9

H. Physical Functioning

0 71 62.9 2.4 72 65.5 2.8 134 64.9 1.9

6 70 64.4 2.9 71 66.2 2.7 130 65.7 2.1

12 70 64.1 3.1 71 66.0 2.7 133 64.6 2.1

18 70 69.4 2.8 71 66.9 2.9 133 65.6 2.2

24 69 66.2 2.8 70 66.4 2.8 133 65.3 2.1

I. Social Functioning

0 71 67.1 3.0 72 63.0 3.1 134 67.5 2.2

6 70 73.5 3.1 71 68.2 3.1 130 68.0 2.3

12 70 72.4 3.0 71 66.7 3.2 133 69.7 2.2

18 70 78.1 2.9 71 67.0 3.4 133 69.6 2.6

24 69 74.7 3.1 70 67.9 3.1 133 71.2 2.4

J. Role‑Physical

0 71 41.5 4.9 72 54.9 5.0 134 45.7 3.7

6 70 57.1 5.0 71 62.3 4.9 130 51.5 3.6

12 70 56.1 4.9 71 47.2 5.0 133 49.4 3.8

18 70 60.4 4.8 71 51.8 5.1 133 50.0 3.8

24 69 53.3 5.5 70 51.4 4.9 133 48.5 3.7

K. Role‑Mental

0 71 71.4 4.7 72 69.9 4.6 134 66.7 3.5

6 70 70.5 4.5 71 68.5 4.4 130 68.2 3.3

12 70 69.5 4.4 71 52.1 5.1 133 62.9 3.7

18 70 71.0 4.7 71 53.5 5.1 133 68.2 3.4

24 69 77.3 4.3 70 62.4 5.1 133 61.9 3.8

**High Responders Low Responders Placebo**

**Months** N Mean SE N Mean SE N Mean SE

L. Mental Health

0 71 73.6 1.9 72 69.6 2.1 134 71.3 1.7

6 70 73.8 2.0 71 67.4 2.4 130 70.3 1.6

12 70 72.4 2.3 71 68.7 2.4 133 71.9 1.7

18 69 73.0 2.3 71 66.8 2.5 133 71.2 1.8

24 69 75.4 2.3 70 66.6 2.3 133 71.2 1.7

M. Energy‑Fatigue

0 71 48.9 2.2 72 47.6 2.3 134 48.4 1.9

6 70 53.1 2.5 71 51.5 2.2 130 49.3 1.9

12 70 53.1 2.5 71 51.7 2.5 133 50.9 1.8

18 70 53.0 2.5 71 54.2 2.6 133 50.0 2.1

24 69 53.8 2.7 70 51.4 2.4 133 51.0 1.9

N. Pain Recall (4‑week)

0 71 46.6 2.7 72 46.8 3.1 134 46.3 2.2

6 70 56.7 2.8 71 50.1 3.0 130 48.6 2.4

12 70 58.3 3.2 71 49.8 3.0 133 49.6 2.4

18 70 60.3 3.0 71 50.2 3.1 133 47.1 2.4

24 69 57.6 3.2 70 48.6 3.0 133 49.5 2.3

##### 
